# Utility of a bone health clinic in bridging the osteoporosis care gap: Prescribing habit review at an academic institution

**DOI:** 10.1371/journal.pone.0307029

**Published:** 2024-07-18

**Authors:** Marisa Riley, Derek Crossman, Paul Kocis, Susan Hassenbein, Edward Fox

**Affiliations:** 1 Penn State College of Medicine, Hershey, Pennsylvania, United States of America; 2 Department of Pharmacology, Penn State College of Medicine, Hershey, Pennsylvania, United States of America; 3 Department of Orthopaedics and Rehabilitation, Penn State College of Medicine, Hershey, Pennsylvania, United States of America; Federal University of Minas Gerais: Universidade Federal de Minas Gerais, BRAZIL

## Abstract

**Objective:**

To analyze osteoporosis medication prescribing trends across specialties in the context of a Bone Health Clinic.

**Introduction:**

Osteoporosis affects over 10 million adults in the US, taking a significant toll on patients and the healthcare system. Although screening methods and treatments are improving, the disease remains underdiagnosed and undertreated. This study aims to evaluate the prescribing trends of osteoporosis medication among department specialties to delineate the benefits of a bone health clinic.

**Methods:**

Retrospective data collection identified and analyzed patients within the Penn State Health system prescribed one of the following osteoporosis medications: Bisphosphonate, denosumab, romosozumab, teriparatide, abaloparatide, or raloxifene. Date range: 4/18/2016 to 4/14/2021. Data collection identified the specialty origin of prescriptions for osteoporosis medications across various medical specialties (e.g., orthopaedics, family medicine, and internal medicine).

**Results:**

10,736 prescription orders were issued to patients with an average age of 68 years. Non-Hispanic Caucasian patients received 88.6% of prescriptions, followed by Asian (3.4%) and African American (2.2%). Female patients accounted for 87.8% of all prescriptions. The Bone Health Clinic under two orthopaedic providers wrote 3,619 prescriptions, averaging 361.9 prescriptions per provider per year—marking the highest rate among specialties. The clinic prescriptions constituted 33.7% of all prescriptions across specialties. Orthopaedic surgery prescribed the most denosumab, romosozumab, teriparatide, and abaloparatide prescriptions, and had the highest number of male osteoporosis patients compared to other specialties (15.6%), consequently prescribing the most male prescriptions (578).

**Conclusion:**

Establishing a bone health clinic dedicated to osteoporosis management leads to significantly higher prescription rates per provider, increased utilization of anabolic therapies compared to other specialties, and more male patients being treated—an often-neglected population in osteoporosis.

## Introduction

An estimated 10.2 million adults in the United States have osteoporosis, with an additional 43.3 million having low bone mass [1]. One of the most common and debilitating manifestations of osteoporosis is the fragility fracture, often serving as a sentinel event with severe morbidity and mortality consequences while also burdening the Healthcare system. The projected annual incidence of fractures is expected to rise from 1.9 million to 3.2 million (a 68% increase) between 2018 and 2040 [2–4]. Osteoporotic fractures precipitate more hospitalizations than heart attacks, strokes, and breast cancer, with most patients remaining untreated for their underlying bone disease and at high risk for subsequent fractures [5–7]. Despite the prevalence and severe repercussions of osteoporosis, it remains underdiagnosed and undertreated [6, 8].

As there is no cure for osteoporosis, the main goal of treatment is primary and secondary fracture prevention through bone health optimization. Despite the presence of established screening criteria and several treatment options, studies show that a large portion of patients susceptible to life-altering fractures do not receive adequate management for osteoporosis. Additionally, treated patients may receive sub-optimal care [9, 10].

The persistent and multifactorial gap in fragility fracture management is related to treatment initiation failure and inadequate adherence to treatment despite the continued development of evidence-based guidelines [10, 11]. Barriers to implementing effective osteoporosis care include cost, medication hesitancy, medical comorbidities, polypharmacy, dementia, aging, and the need for clarity on which specialty is responsible for managing these patients [9, 12–14].

Several specialties, including orthopaedic surgery, family medicine, internal medicine, endocrinology, and rheumatology, can effectively manage the patient population. The disease’s indolent nature makes it a low priority during office or hospital visits, allowing the extent of damage to go unrecognized until a sentinel fracture occurs [14]. As a result, an orthopaedic surgeon is often the first physician to see osteoporosis patients and address their underlying bone disease directly. However, this relationship is usually limited to follow-up as an outpatient, and many surgeons believe primary care providers should be responsible for evaluating and treating osteoporosis [8, 15, 16]. The ambiguity regarding who manages osteoporotic patients increases the care gap.

The prevalence of osteoporosis will increase as the human population increases and exact major economic and human tolls [2, 17, 18]. Health care must adapt and devise new solutions to enhance the treatment rates of patients with osteoporosis and alleviate the global care gap. One strategy involves implementing a designated bone health clinic tailored to identify and treat vulnerable patients and pursue longitudinal follow-up, which is otherwise neglected. A bone health clinic dedicated to osteoporosis management could give its providers the ability to increase prescription rates and administer more specialized, long-term care for patients. These strategies reduce ambiguity related to specialty or provider [4, 19–23].

This study employs descriptive and comparative statistics to evaluate the prescribing patterns of osteoporosis medications at the Penn State Hershey Medical Center (PSHMC) Orthopaedic Bone Health Clinic and compare the trends to those of other PSHMC department specialties. We hypothesize that a Bone Health Clinic dedicated to osteoporosis care will significantly contribute to the total prescription count and have the highest rate of prescriptions per provider per year compared to other specialties. This study’s qualifying osteoporosis diagnosis includes all primary and secondary types. This study does not stratify or analyze patient populations based on the type of medication received.

This study quantifies the benefits of a Bone Health Clinic for osteoporosis care within one health system by comparing the volume of prescriptions, the class of medications prescribed, and the demographics of the patients treated. No other paper thus far has described the prescribing habits of specialties treating osteoporosis or, furthermore, those habits in the context of a bone health clinic.

## Methods

### Establishing the Bone Health Clinic

The Bone Health Clinic within the orthopedic surgery department at Penn State University is championed by an orthopedist with departmental leadership support, both deeming osteoporosis care as a prerogative. At other institutions, the champions in bone health have been demonstrated in either endocrinology, rheumatology, or other subspecialties [19, 24].

Two orthopaedic providers run the Bone Health Clinic: one orthopaedic surgeon and one nurse practitioner. No other providers included in this study were associated with the clinic. Every patient was eligible for referral, regardless of fracture status, via the Penn State Health System and external community referrals, most commonly through internal medicine and family medicine. For this clinic, the attending physician and nurse practitioner evaluate the referrals, and if available, all records are sent to the clinic before consultation, including laboratory studies and DEXA scans. If not available, these studies are ordered at the time of the initial consultation.

### Data collection

This study was determined to be exempt from the requirement for informed consent by The Human Subjects Protection Office at Penn State University, in accordance with institutional policies and relevant federal regulations. Participant anonymity and confidentiality were ensured throughout the study, and no identifiable information was collected.

For this retrospective chart review, patient cohort data were identified through TriNetX and aggregate patient data from Penn State Health. Patients were acquired through a request made to the Penn State Health Clinical and Translational Science Institute (CTSI). Subject inclusion criteria consisted of males/females ≥ 18 years of age (non-pregnant) and receiving a prescription for one of the following osteoporosis medications: bisphosphonate, denosumab, romosozumab, teriparatide, abaloparatide, or raloxifene. The date range for inclusion was from April 18, 2016, to April 14, 2021.

The prescribing habits of the Bone Health Clinic and medical specialties were analyzed through descriptive statistics to compare medication prescribing trends between the clinic and different specialties within one health system. Several qualitative variables, including drug class, drug and provider counts, and most demographic parameters, were included in this analysis. Additionally, age differences across specialties were assessed using comparative statistics through one-way analysis of variance (ANOVA) and post-hoc pairwise comparisons employing the Tukey-Kramer method, all conducted using SAS software, version 9.4.

## Results

Among the 10,736 prescription orders identified, the average patient age was 68 years. Significant differences were observed in the average age of patients between the Bone Health Clinic (66) and family medicine (71.6) (p < 0.001), as well as the clinic and internal medicine (71.6) (p < 0.001). Non-Hispanic Caucasian patients accounted for 88.6% of all prescriptions, followed by Asian patients at 3.7% and African American patients at 2.2%. The remaining prescriptions were distributed among various other demographic groups. Additionally, 2.7% of prescriptions were for patients who ethnically identified as Hispanic, Latino, or Spanish origin. Female patients received the majority of prescriptions, comprising 87.8% of the total.

Twenty-two prescription orders for metastatic cancer dosing drug regimens involving zoledronic acid (4 mg) or denosumab (120 mg) were excluded from the analysis and are not included in the 10.736 total prescriptions. The remaining 3.3% of patients with a cancer diagnosis received osteoporotic drug doses and were included in the results.

**Fig 1:**
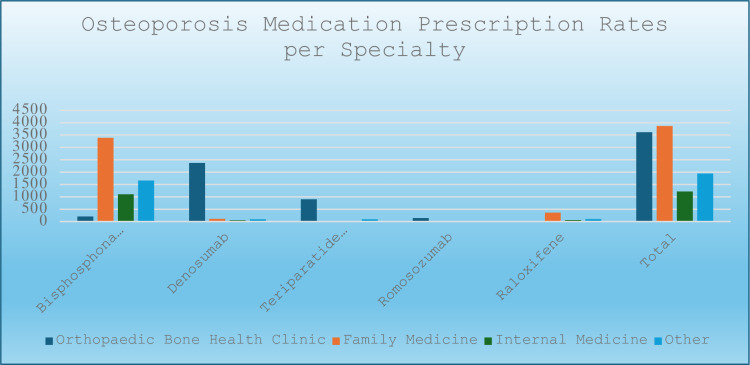
Prescription rates of osteoporosis medications among Penn State Health medical specialties and subspecialties.

Outside of the two orthopaedic providers (the author and his nurse practitioner) specifically associated with the Bone Health Clinic, eight other orthopaedic surgery providers prescribed one or more osteoporosis medications: one sports medicine physician, one nurse practitioner, four physician assistants, and two resident physicians. Collectively, the orthopaedic group accounted for 3,714 prescriptions, representing 34.5% of all written prescriptions (Fig 1).

**Fig 2:**
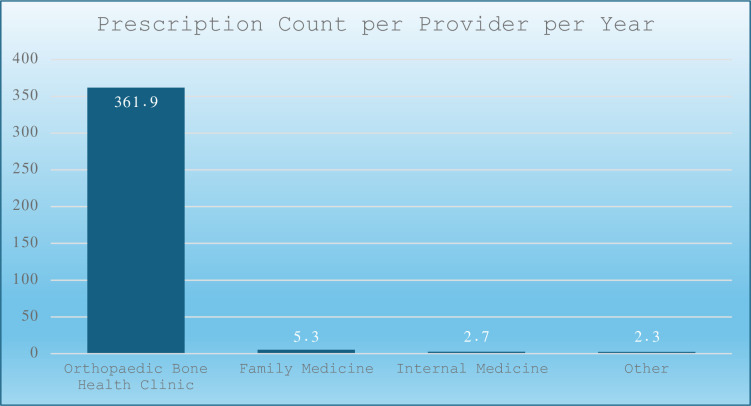
The average prescription count per provider per year among Penn State Health medical specialties and subspecialties.

Notably, the Orthopaedic Bone Health Clinic alone accounted for 3,619 prescriptions, or 97.4% of the department’s total, with an average of 361.9 prescriptions per provider per year (Fig 2).

**Fig 3:**
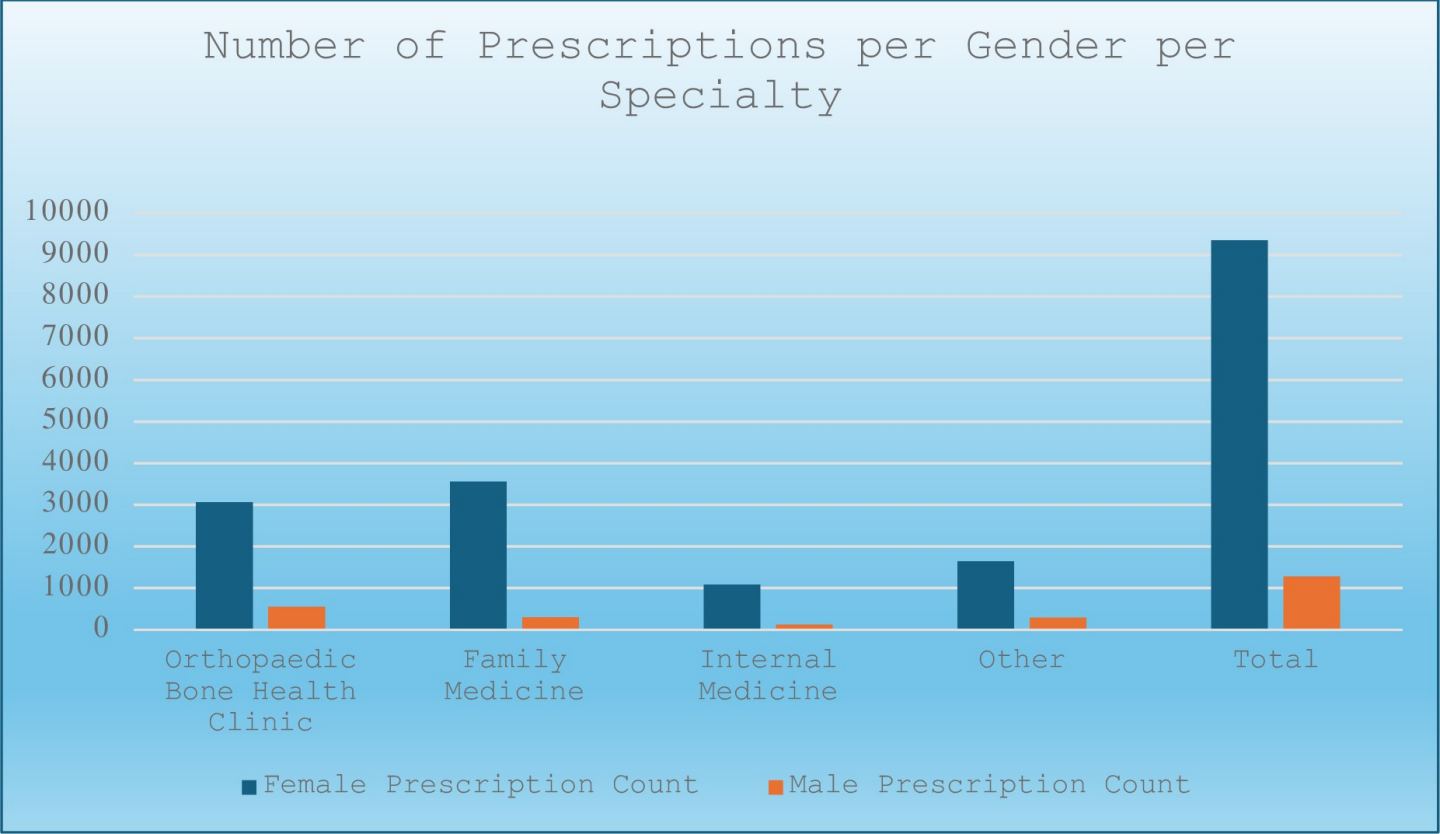
Prescribing trends categorized by gender and the number of prescriptions ordered by different specialties. Of the 10,736 entries, 9,430 were female and 1,306 were male.

The bone health clinic prescribed 32.5% of all female prescriptions (9430) and 42.6% of all male prescriptions (1306) (Figs 1 and 3). Orthopaedic surgery had the highest proportion of prescriptions for male patients at 15.6%, compared to 7.9% in family medicine and 10.2% in internal medicine.

Denosumab was the most frequently prescribed medication by the Bone Health Clinic, accounting for 65.5% of prescriptions and representing 88.5% of all denosumab orders across specialties (Fig 1). Other commonly prescribed medications in the clinic included teriparatide (15.6%), abaloparatide (9.2%), alendronate (5.3%), and romosozumab (3.9%). Romosozumab was predominantly prescribed by the Bone Health Clinic, totaling 142 prescriptions, with only one prescription issued by family medicine. Furthermore, the Bone Health Clinic accounted for 84.6% of all teriparatide prescriptions and 93.9% of all abaloparatide prescriptions.

**Fig 4:**
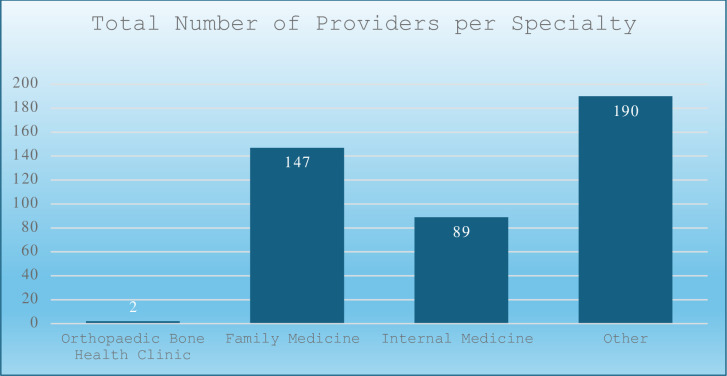
Penn State Health medical specialties and subspecialties with the associated number of providers prescribing osteoporosis medications.

In contrast, family medicine prescribed 3,685 prescriptions through 147 providers, representing 36% of all prescriptions and averaging 5.3 prescriptions per provider per year. The majority (87.7%) of these prescriptions belonged to the bisphosphonate class (Figs 1, 2 and 4).

Similarly, internal medicine wrote 1,215 prescriptions through 89 providers, averaging 2.7 prescriptions per provider per year with a 90.9% bisphosphonate majority.

Rheumatology, endocrinology, and hematology-oncology had the fourth, fifth, and sixth highest prescription rates, respectively. Similar to family medicine and internal medicine, the prescriptions from rheumatology (91.9%), endocrinology (77.7%), and hematology-oncology (91.1%) were primarily bisphosphonates.

Each of the remaining specialties distributed less than 100 prescriptions and factored into the “other” category with endocrinology, rheumatology, and hematology-oncology, totaling 1,942 prescriptions.

## Discussion

Osteoporosis is a prevalent, debilitating disease. Despite the presence of many effective therapies, a clear need exists for innovative programs to improve current osteoporosis management. Currently, many patients who would benefit from treatment fall through the cracks of the healthcare system or receive suboptimal treatment. Effective osteoporosis identification and treatment are necessary to mitigate the profound burden this disease has both on individuals and the healthcare system at large [10].

Our study shows several benefits of implementing a dedicated bone health clinic within an established healthcare system. One of the most striking is the efficacy shown through the markedly elevated average prescription rate per provider per year within the Orthopaedic Bone Health Clinic, which reached 361.9 prescriptions compared to 5.3 for family medicine and 2.7 for internal medicine. The orthopaedic surgeon and nurse practitioner at the Bone Health Clinic administered 97.4% of the total prescription count within the orthopaedic surgery department and 33.7% of all prescriptions across specialties. While family medicine yielded the highest total prescription count (3,865), this was distributed among 147 providers. The efficiency of the Bone Health Clinic is evident in its ability to maintain a substantial prescription output with a minimal number of providers.

Furthermore, the Bone Health Clinic prescribed more denosumab and anabolic agents than all other specialties. Denosumab was the most frequently prescribed drug (65.5%), followed by teriparatide (15.6%), abaloparatide (9.2%), alendronate (5.3%), and romosozumab (3.9%). In contrast, family medicine and internal medicine predominantly prescribed bisphosphonates, with rates of 87.7% and 90.9%, respectively. Ultimately, a dedicated bone health clinic offers a greater variety of therapies compared to other specialties. The clinic is more likely to use newer, more advanced medications, including denosumab, teriparatide, abaloparatide, and romosozumab.

Interestingly, among all the specialties, orthopaedic surgery treated the greatest number of males (578). This finding is noteworthy, given that osteoporosis treatments are primarily focused on female populations despite higher mortality rates related to osteoporosis in men [3, 25]. Nevertheless, osteoporosis remains undertreated in the male population [25, 26].

Our results suggest that establishing a bone health clinic may benefit this under-recognized and vulnerable patient population that harbors over 2 million osteoporosis patients and 16 million additional low bone density patients in the United States [1]. Most other demographic results were consistent across specialties. The author attributes potential biases, detailed later in this discussion, to the age difference between patients at the Bone Health Clinic and family medicine or internal medicine.

Our study was limited in several ways. Data was limited by the information recorded in our database, and although we could identify prescriptions, we could not detail if prescriptions were filled. Since we did not include follow-up data, we could not address compliance with management strategies, report the incidence rate of additional fractures, or determine if there was a transfer of care. Additionally, our study aimed to evaluate prescription trends rather than analyze referral sources, potentially introducing bias in cases where, for example, providers of other specialties automatically shuttled non-alendronate patients to the Orthopaedic Bone Health Clinic. Lastly, we did not identify who initially diagnosed osteoporosis or distinguish pre-fracture patients identified in the clinic from those internally referred by orthopaedics.

Future research analyzing referral sources for osteoporosis patients could assist with interpreting prescription trends, including why a bone health clinic might receive the most non-bisphosphonate patients or those seeking specific therapies like denosumab. Determining which specialties make the initial diagnoses of osteoporosis would also provide further insight into the patient population comorbidities of a bone health clinic. Additionally, studies collecting compliance and follow-up data along with fracture and osteoporosis treatment histories would provide information regarding the Bone Health Clinic’s efficacy in management and impact on the incidence of fragility fractures.

In conclusion, this study demonstrates that a designated bone health clinic successfully fills a gap in osteoporosis care and can play a crucial role in reducing the fracture burden. To further reduce the current burden of osteoporosis, barriers to treatment must be addressed [14, 16, 27]. Our study results can assist health care policy-makers in strategies to allocate resources for this disease in cost-effective ways, such as implementing dedicated bone health clinics and providers with the ultimate goal of enhancing community bone health and decreasing the number of fragility fractures.

## Supporting information

S1 Dataset (XLSX)

S1 File ANOVA. (DOCX)
