# Supplementary material for: Utility of a bone health clinic in bridging the osteoporosis care gap: Prescribing habit review at an academic institution
Source: PLoS One. 2024 Jul 18;19(7):e0307029. doi: 10.1371/journal.pone.0307029 (PMC11257224; doi:10.1371/journal.pone.0307029)
Supplement: S1 File — (DOCX) [file pone.0307029.s002.docx]

| **Overall Test of Fixed Effects** | | | | |
| --- | --- | --- | --- | --- |
| **Effect** | **Num DF** | **Den DF** | **F Value** | **P-value** |
| **Group** | 2 | 8696 | 280.62 | <.0001 |

| **Group** | **Estimated Marginal Mean (95% Confidence Interval)** |
| --- | --- |
| Family Med | 71.64 (71.30, 71.98) |
| Internal Med | 71.61 (71.00, 72.22) |
| Ortho BHC | 66.06 (65.71, 66.41) |

|  | **Unadjusted for Post-hoc Pairwise Comparisons** | | **Adjusted for Post-hoc Pairwise Comparisons using the Tukey-Kramer Method** | |
| --- | --- | --- | --- | --- |
| **Comparison** | **Difference in Estimated Marginal Means (95% Confidence Interval) [Unadjusted]** | **P-value [Unadjusted]** | **Difference in Estimated Marginal Means (95% Confidence Interval) [Adjusted]** | **P-value [Adjusted]** |
| (Family Med) vs. (Internal Med) | 0.03 (-0.67, 0.72) | 0.943 | 0.03 (-0.81, 0.86) | 0.997 |
| (Family Med) vs. (Ortho BHC) | 5.58 (5.09, 6.07) | <.001 | 5.58 (4.99, 6.16) | <.001 |
| (Internal Med) vs. (Ortho BHC) | 5.55 (4.85, 6.25) | <.001 | 5.55 (4.71, 6.39) | <.001 |
